# The complete chloroplast genome of greater duckweed (*Spirodela polyrhiza* 7498) using PacBio long reads: insights into the chloroplast evolution and transcription regulation

**DOI:** 10.1186/s12864-020-6499-y

**Published:** 2020-01-28

**Authors:** Yating Zhang, Dong An, Changsheng Li, Zhixuan Zhao, Wenqin Wang

**Affiliations:** 1 School of Agriculture and Biology, Shanghai Jiao Tong University, Shanghai, China; 2 National Key Laboratory of Plant Molecular Genetics, CAS Center for Excellence in Molecular Plant Sciences, Institute of Plant Physiology & Ecology, Shanghai Institutes for Biological Sciences, Chinese Academy of Sciences, Shanghai, China

**Keywords:** Duckweeds, Chloroplast genome, PacBio, Intron, RNA editing, Operon

## Abstract

**Background:**

Duckweeds (*Lemnaceae*) are aquatic plants distributed all over the world. The chloroplast genome, as an efficient solar-powered reactor, is an invaluable resource to study biodiversity and to carry foreign genes. The chloroplast genome sequencing has become routine and less expensive with the delivery of high-throughput sequencing technologies, allowing us to deeply investigate genomics and transcriptomics of duckweed organelles.

**Results:**

Here, the complete chloroplast genome of *Spirodela polyrhiza* 7498 (SpV2) is assembled by PacBio sequencing. The length of 168,956 bp circular genome is composed of a pair of inverted repeats of 31,844 bp, a large single copy of 91,210 bp and a small single copy of 14,058 bp. Compared to the previous version (SpV1) assembled from short reads, the integrity and quality of SpV2 are improved, especially with the retrieval of two repeated fragments in *ycf2* gene. There are a number of 107 unique genes, including 78 protein-coding genes, 25 tRNA genes and 4 rRNA genes. With the evidence of full-length cDNAs generated from PacBio isoform sequencing, seven genes (*ycf3*, *clpP*, *atpF*, *rpoC1*, *rpl2*, *rps12* and *ndhA*) are detected to contain type-II introns. The *ndhA* intron has 50% more sequence divergence than the species-barcoding marker of *atpF-atpH*, showing the potential power to discriminate close species. A number of 37 RNA editing sites are recognized to have cytosine (C) to uracil (U) substitutions, eight of which are newly defined including six from the intergenic regions and two from the coding sequences of *rpoC2* and *ndhA* genes. In addition, nine operon classes are identified using transcriptomic data. It is found that the operons contain multiple subunit genes encoding the same functional complexes comprising of ATP synthase, photosynthesis system, ribosomal proteins, et.al., which could be simultaneously transcribed and coordinately translated in response to the cell stimuli.

**Conclusions:**

The understanding of the chloroplast genomics and the transcriptomics of *S.polyrhiza* would greatly facilitate the study of phylogenetic evolution and the application of genetically engineering duckweeds.

## Background

*Lemnaceae* (duckweeds) are the fastest growing plants including five genera of *Spirodela*, *Landoltia, Lemna*, *Wolffiella* and *Wolffia*. They are phylogenetically located at the early-diverging monocots of the *Alismatale* order. Duckweeds have ecological and economical merits as wastewater treatment, animal feed and biofuel. The morphology is extremely simplified and small, resulting in the difficulty of species or ecotypes identification [1, 2]. The chloroplast genome has dual characteristics of sequence variation and conservation, which are widely applied in the studies of population genetics and phylogenetic relationships. The entire chloroplast genomes show the potential to serve as a plant super-barcode to distinguish closely related species such as in Conyza (in the family of Asteraceae) [3, 4] and *Theobroma cacao* (in the family of Malvaceae) [5]. The chloroplast genome is one of the three genetic systems including nuclei, mitochondria, and plastids in plants that possesses both eukaryote-like introns and prokaryote-like operons [6]. One broad hypothesis is that the chloroplast is derived from an initial engulfment and integration of a free-living cyanobacterium into a host cell around 1.5 billion years ago [7]. Group I and II introns in chloroplasts and mitochondria are a large class of self-catalytic ribozymes either with or without assistance from proteins for vivo splicing. In particular, group II introns have the ability of retrotransposition through intron-encoded reverse transcriptase activities [8]. Although most ancestral genes were transferred into the host nucleus during chloroplast evolution, modern chloroplast genomes possess common structural features with a size of ~ 107–218 kb and are compacted with a gene content of ~ 100–120 genes [9]. The chloroplast is also a vital organelle for plants, playing a crucial role by converting solar energy to carbohydrates through photosynthesis, and promoting their growth and starch accumulation.

With the rapid development of sequencing technology, it is easier and cheaper to obtain the complete genomes including nuclei, mitochondria and chloroplast [10]. In 2008, the first duckweed chloroplast genome (*L.minor*) was sequenced by Sanger sequencing [11]. Another three chloroplast genomes (*S.polyrhiza* 7498, *W.lingulate* 7289, and *W.australiana* 7733) were sequenced by using the SOLiD platform generating short reads (~ 50 bp) and assembled in 2011 [12]. The recent eight species covered the genera of *Landoltia*, *Lemna* and *Wolffia* were assembled by using the Illumina platform to study duckweed phylogeny [13]. In the meanwhile, the duckweed nuclear genomes have become more complete with the expansion of sequencing technology. The *Spirodela* nuclear genomes were generated by physical mapping and short-read DNA sequencing strategies [14, 15]. The *Spirodela* genome has continued to be improved by integrating the evidences from cytogenomic, optical mapping and Nanopore sequences [16]. Long-read sequencing, such as SMRT (Single Molecule Real-Time) technology emerged in 2009 [17] has been widely applied in sequencing the chloroplast genomes with the improved contiguity and accuracy. Still, no duckweed chloroplast genomes based on long-read sequencing have been reported. The studies of annotating chloroplast genome and gene structure at the transcriptomic and post-transcriptomic levels were limited, which were involved in a series of RNA regulation and process, such as RNA splicing, 5′- and 3′-end modification, and RNA editing and turnover [18]. Most previous studies relied on the sequence alignment and computer prediction to determine the intron boundary and the possible RNA editing sites, which need to be confirmed by PCR and sequenced one by one [19, 20]. With the high-throughput RNA-seq data with a read length of 75 bp, 66 RNA editing in *Spirodela* chloroplast genome were defined at the genome-wide level [21]. However, such short reads of 75 bp were impossible to accurately set intron and exon boundaries, as well as to distinguish the operons without the full-length cDNA sequences.

Here, we initiated a project that was originally designed as the nuclear genome sequencing and annotation by using long PacBio reads [22]. Since the raw reads were generated from the total DNA and RNA, we took advantage of such data to study chloroplast genomics and transcriptomics. In this study, we improved and validated the chloroplast genome of *S.polyrhiza* assembled by PacBio sequencing reads with retrieval of two repeated fragments compared with the last version. The integration of full-length cDNAs from isoform sequencing allowed us to discover new RNA editing sites, to detect introns, and to define poly-cistrons similar to prokaryotic transcripts in *Spirodela* chloroplast. The understanding of the chloroplast genomics and the transcriptomics of *S.polyrhiza* would facilitate the study of phylogenetic evolution and the application of genetically engineering the solar reactor of chloroplasts.

## Results

### Chloroplast genome assembly, validation and annotation

**Fig. 1:**
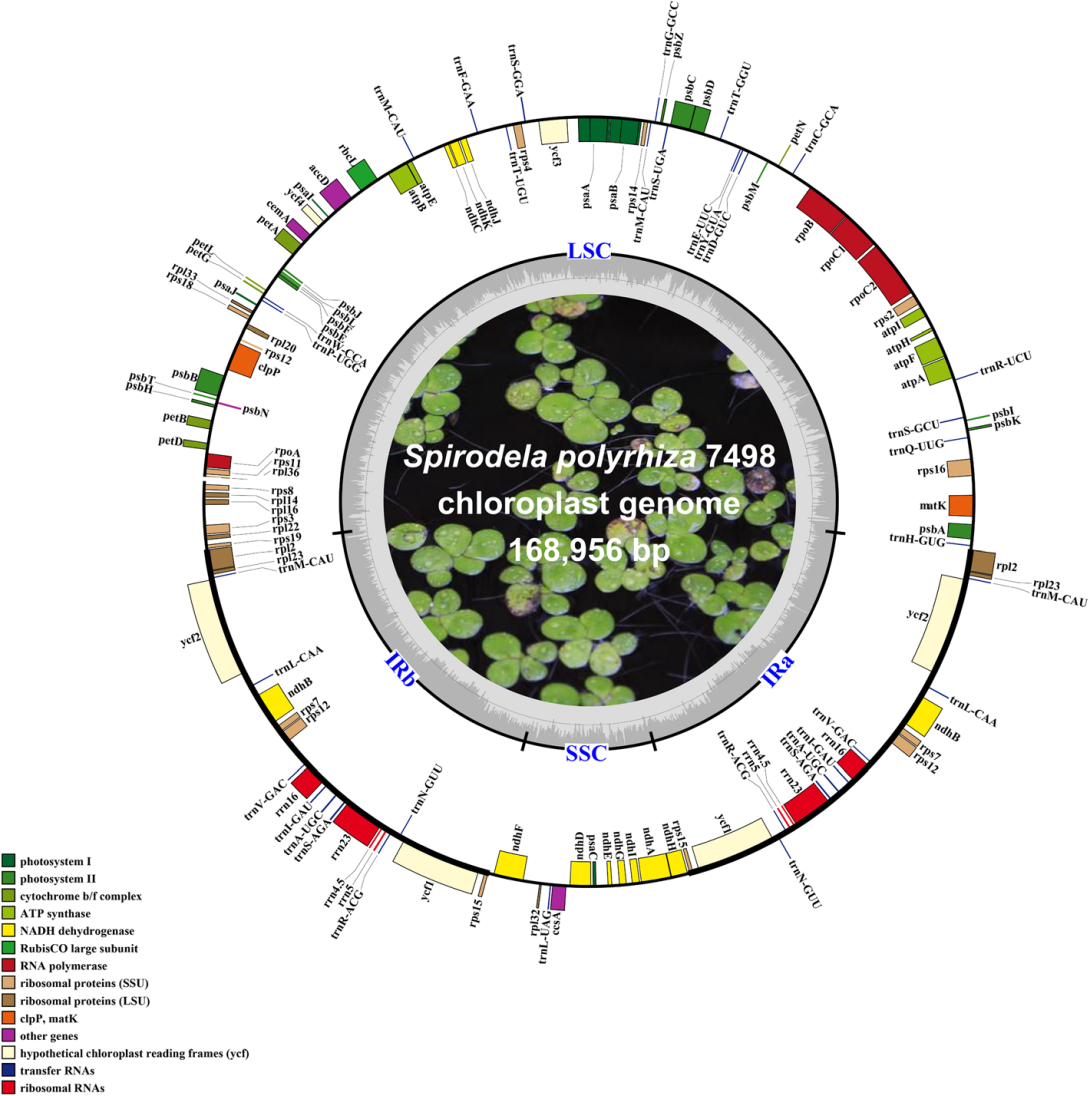
Gene map of the chloroplast genome of *S.polyrhiza* 7498. Genes are labelled based on the annotation data. Genes are color-coded in different functional groups. The middle circle indicates a quadripartite structure. The darker area in the inner circle indicates the GC content

**Fig. 2:**
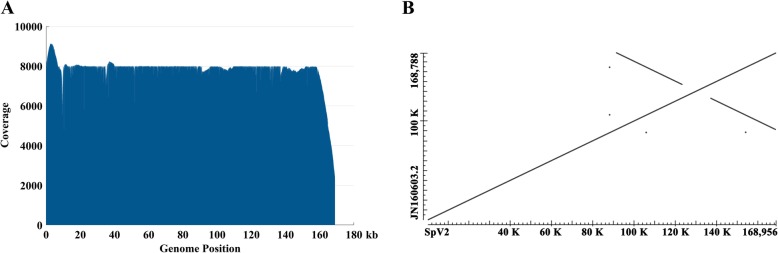
Sequencing coverage and genome comparison. **a** The x-axis shows the chloroplast genome of *S.polyrhiza*. The y-axis indicates the sequencing depth across the genome. **b** The sequence alignment of two versions of *S.polyrhiza* 7498 chloroplast genomes. The lines indicate the genome collinearity and IR regions

The last version of the complete chloroplast genome of *S.polyrhiza* 7498 (SpV1) was sequenced on a SOLiD platform and published in 2011 (GenBank accession number: JN160603) [12]. Because of the limitations of the second-generation sequencing technology with short reads (50 bp), the assembly of SpV1 was tedious and challenging to resolve boundaries of IR regions, resulting in 3 genomic breakage and 52 small gaps (Table 1). Here, the total DNA originated from nuclei, mitochondrion and chloroplasts was prepared from the whole duckweed tissue using CTAB method [23]. The high-quality DNA was sequenced on the PacBio platform, generating long reads with the mean length of 10,789 bp. After bioinformatic filtering, a total of 239,086 high-quality long reads were selected to be chloroplast related sequences, which were used to run the chloroplast genome de novo assembly. A single circular strand genome with a size of 168,956 bp (GenBank accession number: MN419335) was directly constructed by using a long-read based bioinformatic pipeline (Additional file 1: Figure S1) [24] without any manual correction and sequence collapses, skipping further PCR amplification and capillary electrophoresis (CE) sequencing to fill unassembled gaps. In contrast, SpV1 was assembled from short reads with a read length of 50 bp, resulting in 52 contigs and 3 scaffolds (Table 1). The broken scaffolds were manually ordered based on other chloroplast genomes. A number of 52 pairs of primers were designed to close the gaps and to reach the final genome with tremendous efforts [12]. The chloroplast genome with long-read assembly exhibited the typical quadripartite structure, a pair of inverted repeat regions (IRs) of 31,844 bp separated by a large single copy (LSC) of 91,210 bp and a small single copy (SSC) of 14,058 bp (Fig. 1). The GC content was 40.06, 33.47 and 30.17%, respectively, and the overall GC content was 35.68%. The sequence similarity between SpV2 and SpV1 was 99.9% (Fig. 2), indicating high accuracy of the assembled genome. The chloroplast genome was annotated as 107 unique genes, including 78 protein-coding genes, 25 tRNAs and 4 rRNAs. There were 19 genes, including seven protein-coding genes, eight tRNAs and four rRNAs in the IR regions (Additional file 1: Table S1). A coverage plot was demonstrated by re-mapping the PacBio reads to the chloroplast genome, showing an even distribution across the genome with a mean coverage of 7837 times (Fig. 2).

**Table 1:** The comparative statistics of the chloroplast genome assembly of *S.polyrhiza* 7498 generated from long reads of PacBio and short reads of SOLiD platform

Category	PacBio	SOLiD
Number of selected reads^a^	239,086	19,906,092
Total nucleotides (selected data) (bp)^a^	2,579,414,638	995,304,600
Mean read length (selected data) (bp)^a^	10,789	50
Number of scaffolds	1	3
Number of genome gaps	0	52
Total genome coverage	7837	5474
Genome Size (bp)	168,956	168,788
LSC (bp)	91,210	91,222
SSC (bp)	14,058	14,056
IR (bp)	31,844	31,755
GC content (%)	35.68	35.69
GenBank ID	MN419335	JN160603

^a^Only the selected chloroplast-related PacBio reads and SOLiD reads are counted

**Fig. 3:**
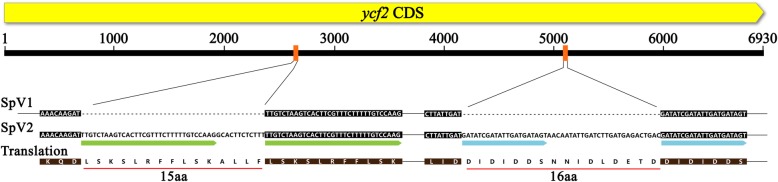
The comparison of *ycf2* gene in SpV1 and SpV2. The *ycf2* gene in SpV2 are 6930 bp, containing two sets of repeats labelled with green and blue arrow, while one copy of repeats is missing in SpV1 due to the limitation of short-read assembly

*Ycf2* was a large functional gene encoding 2310 amino acids in chloroplast IR regions. We retrieved two extra fragments of 45 bp and 48 bp which were located at 2599 and 5065 bp within *ycf2* gene compared to the previous version (Fig. 3). Surprisingly, the recovered sequences were the copies of the downstream nucleotides, which could be a failure of genome assembly in SpV1 due to short reads of second-generation sequencing. Such limitation could be easily conquered by the nature of PacBio long reads with the spanning of the ambiguous repeats.

### Intron identification

**Fig. 4:**
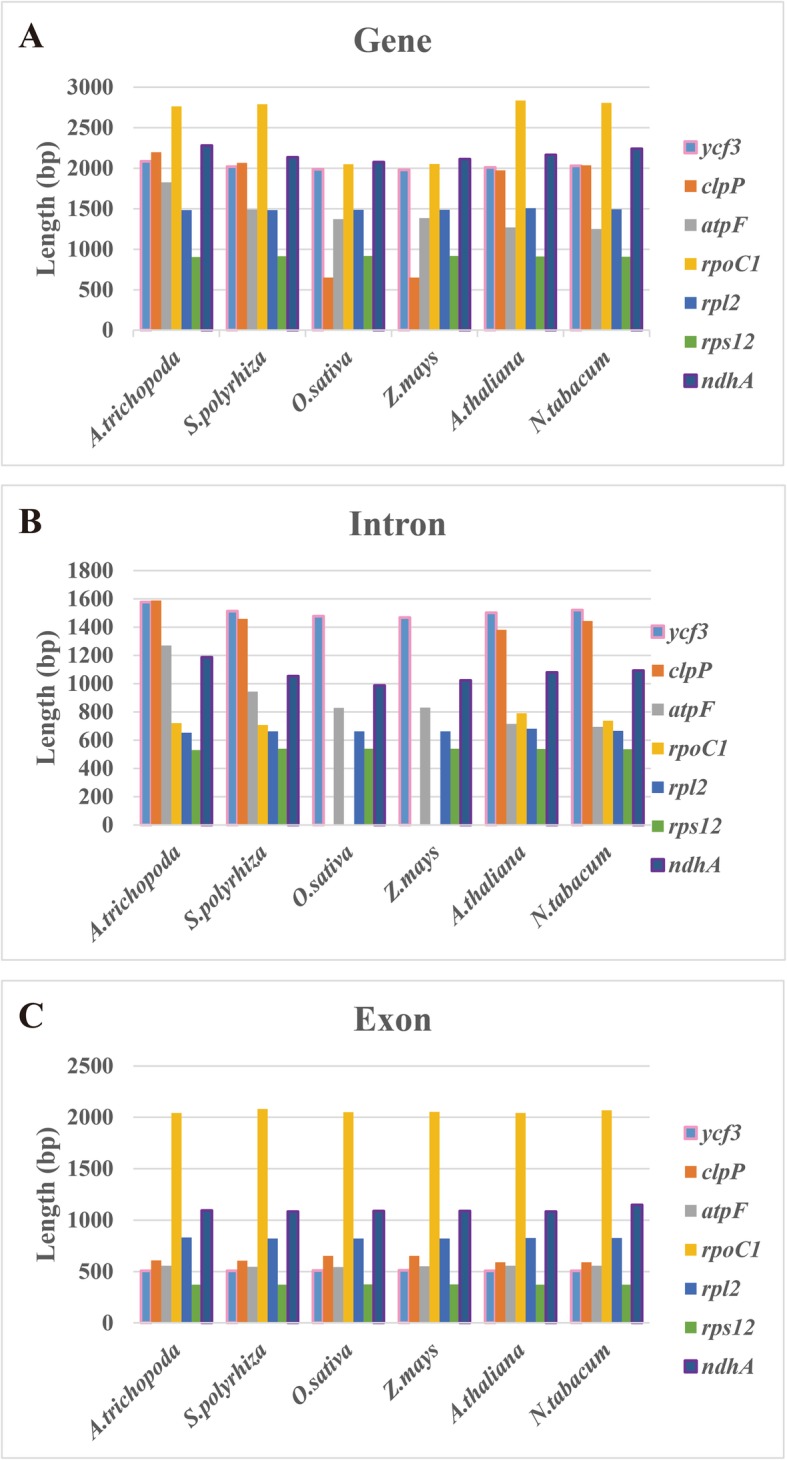
Intron comparison of seven genes in plants. **a**, **b** and **c** display the length of genes, introns and exons within six plant species, respectively. Their sequences are downloaded from *A.trichopoda* (NC_005086.1), *S.polyrhiza* 7498 (MN419335), *O.sativa* (NC_001320.1), *Z.mays* (NC_001666.2), *A.thaliana* (NC_000932.1) and *N.tabacum* (NC_001879.2). The X axis indicates species and Y axis shows sequence length (bp)

The full-length cDNAs generated by PacBio isoform sequencing allowed us to define the chloroplast transcript structures. Here, we defined nine type-II introns within seven genes (*ycf3*, *clpP*, *atpF*, *rpoC1*, *rpl2*, *rps12* and *ndhA*), and the gene of *ycf3* and *clpP* contained 2 introns (Additional file 1: Table S2). We found that the length of introns was extremely conserved in plant species, except the genes of *clpP* and *rpoC1* in *Poaceae* were absent of introns. Previous research has revealed that the intron loss of *rpoC1* and *clpP* genes occurred before grasses species differentiation [25]. We found that the early-diverging monocot of *Amborella* had the longest *atpF* introns (1825 bp), whereas the dicot of tobacco had the shortest one (1250 bp), indicating that introns might play roles in genomic diversity during the chloroplast evolution (Fig. 4). To assess the degree of DNA polymorphism between introns, sequence divergences in four duckweed species were calculated with the overall mean distance respectively. The region of *ndhA* intron showed the highest genetic distance, while the non-coding intron in the *rps12* gene was the most conserved one (Table 2). The *ndhA* intron had 50% more polymorphism compared to the proposed species barcode marker of *atpF*-*atpH* [26], showing sufficient genetic distance and potential to discriminate close species.

**Table 2:** Measurement of intron divergences between duckweed species

Gene	Aligned Length (bp)	Base Variable	Overall Mean Distance
*atpF-atpH*^a^	493	85	0.0960
*rbcL*^b^	1461	92	0.0366
*atpF*	949	147	0.1089
*rpoC1*	740	94	0.0716
*rps12*	540	5	0.0053
*rpl2*	664	8	0.0071
*ndhA*	1091	235	0.1413
*ycf3_1*	778	72	0.0551
*ycf3_2*	827	72	0.0503
*clpP_1*	868	122	0.0875
*clpP_2*	688	94	0.0861

Aligned length are longer than the original sequence length because of the addition of the aligned gaps. Base variation is the base polymorphism excluding insertions or deletions. The controls of the intergenic region of *atpF-atpH*^a^ and the coding sequence of *rbcL*^b^ are also included. The duckweed species include *S.polyrhiza* (MN419335), *L.minor* (DQ400350), *W.ligulata* (JN160604) and *W.australiana* (JN160604)

### RNA editing analysis

After a chloroplast mRNA molecule is transcribed, it usually undergoes RNA editing, a process of C-to-U conversion at specific sites to regulate gene expression and translation in chloroplasts. Here, with isoform sequences, we defined 37 RNA editing sites, including 30 sites that occurred in protein-coding sequences, one in intron and six in non-coding regions (Additional file 1: Table S3). The RNA editing efficiency had a range of 21 to 100% with a median value of 93%. In 2011, the study using Illumina short reads was able to define 66 editing sites [21], 29 of which were overlapped with this study. Combined with known and newly discovered RNA editing sites, there were 74 in total, 62 of which occurred in gene regions, whereas the *Ndh* gene showed the most heavily edited sites (33 sites) (Additional file 1: Figure S2). The eight newly defined editing events contained two from the coding regions of *rpoC2* and *ndhA* genes and six from the location of intergenic regions (Additional file 1: Table S3). The event of RNA editing in *Spirodela rpoC2* was consistent with rice and tobacco, whereas the C-to-U conversion in *ndhA* made *Spirodela* keep the conserved amino acid of L as other plants (Additional file 1: Figure S3).

### Operon classification

**Fig. 5:**
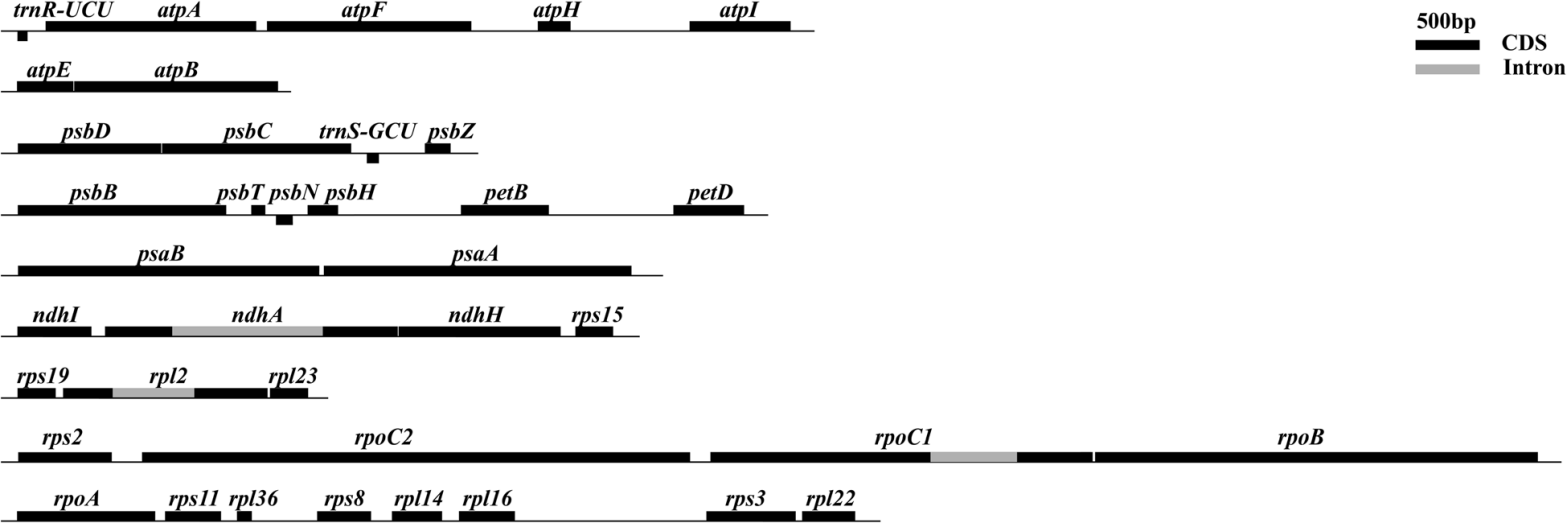
The defined operon structure in *S.polyrhiza* chloroplast. The genes are shown in thick lines with exon in black and intron in grey. The gene names are given on the top of lines. The gene order is based on the physical genome location

An operon, i.e., poly-cistronic mRNA is a messenger RNA that could efficiently encode more than one protein. Such a phenomenon is typical in prokaryotic organisms, including chloroplast due to its origin of cyanobacteria [27]. The coding sequences within an operon is usually grouped and regulated together controlled by a regulatory region of a promoter and an operator. These protein products have a related function of either subunit of building a final complex protein or participating in a common biological process. Thanks to the isoform sequencing with a read length of 10 Kb, we could investigate the operon structures based on the full-length transcripts. Here, we identified nine operons after we mapped transcripts against the genome with a deep coverage (Table 3 and Fig. 5). The operons included gene clusters that encoded different functional groups, such as ATP synthase, RNA polymerase, photosystem II, photosystem I, cytochrome complex, NADH dehydrogenase, ribosome proteins, which are involved in the process of photosynthesis and respiration. It was reported that the *psbB* operon contained genes for the PSII (*psbB*, *psbT*, *psbH*) and cytochrome (*petB* and *petD*) complexes, which are required during chloroplast biogenesis [28]. The enzyme of plastid-encoded RNA polymerase (PEP) was composed core subunits (including the plastid genes of *rpoA*, *rpoB*, *rpoC1* and *rpoC2*) and mainly responsible for the transcription of photosynthesis genes [29, 30]. Like in bacteria and other plants, *rpoA* gene encoding a α-subunit of PEP was found in a gene cluster comprising of ribosomal protein genes in *Spirodela.* The gene cluster of *rpoB*, *rpoC1* and *rpoC2*, encoding the β, β′ and β″ subunits of PEP formed a separate operon (Table 3 and Fig. 5). The operon of NADH dehydrogenase was composed of four genes, mainly involved in electron transport around photosystem I and chloro-respiration. All operons in *Spirodela* had great homology with *Z.mays* and the largest ribosomal protein operon ‘*rpl22-rps3-rpl16-rpl14-rps8-rpl36-rps11-rpoA*’ was consistent with *Cyanophora paradoxa* and *Spinacia oleracea*, where it was called S10 (or spc-like) operon [31, 32]. As we knew, the size of the chloroplast genome was compact, but it played a critical role in photosynthesis in the survival of plants. The pattern of co-transcription in the chloroplast of duckweed may enhance the work efficiency of transcription-translation factors like RNA polymerase.

**Table 3:** The defined operons in SpV2

Operon	Genes	Functions	Length	Genome Position
Atp_1	*atpI*+*atpH*+*atpF*+*atpA*	ATP synthase	5,758	17,612-12,186
Atp_2	*atpB*+*atpE*	ATP synthase	2,141	60,381-58,481
Psb_1	*psbD*+*psbC*+*psbZ*	PSII	3,398	37,462-40,616
Psb_2	*psbB*+*psbT*+*psbH*+*petB*+*petD*	PSII; Cytochrome complex	5,689	78,885-84,218
Psa	*psaA*+*psaB*	PSI	4,818	46,372-41,890
Ndh	*rps15*+*ndhH*+*ndhA*+*ndhI*	NADH dehydrogenase	4,611	137,464-133,111
Rpl_1	*rpl23*+*rpl2*+*rps19*	Ribosomal proteins	2,319	92,997-90,876
Rpo	*rpoB*+*rpoC1*+*rpoC2+rps2*	RNA polymerase; Ribosomal protein	11,837	29,112-17,867
Rpl_2	*rpl22*+*rps3*+*rpl16*+*rpl14*+*rps8* +*rpl36*+*rps11*+*rpoA*	Ribosomal proteins	6,257	90,586-84,434

^a^The length of operon is counted in bp. The column of operon is named with the abbreviation of gene family. The connections of genes are indicated by a plus sign. The gene order in the operon is based on the full-length transcript. Genome Position means the location of operon in the new version of *S.polyrhiza* 7498 chloroplast genome. PSII means photosystem II and PSI is photosystem I

## Discussion

### Third generation sequencing (TGS) technology facilitates chloroplast genomic and Transcriptomic analysis

Compared with second-generation sequencing technologies featured with short reads of 150~300 bp, third-generation sequencing (TGS) has a striking advantage of long reads up to 500 Kb like Nanopore. The long reads could manage repeat regions by using unique flanking sequences and improve genome assembly which can fill potential gaps. Still, the genome completeness depends on the complexity of targeted genomes and the length and quality of sequencing data [10]. With the announcement of the launch of PacBio Sequel II system, it generates 8-times more data and makes sequencing more affordable. No matter how hard scientists try to remove organellar DNA from the total DNA (including nuclear, mitochondria and chloroplast DNA), chloroplast genome still can be assembled from the left “purified” DNA as a side project of the whole genome sequencing study due to its high copy number [33]. Our trial confirmed that two pairs of repeats in the coding sequence of *ycf2* gene were filled in the assembly of the chloroplast genome of *S.polyrhiza*. The phylogenetic analysis suggested that *ycf2* gene was evolved from the membrane-bound AAA-protease *FtsH* of the ancestral endosymbiont [34]. It can be found both in non-green (*Epifagus virginiana*) and green plants, but was absent in the grass family, indicating that its function was not essential for photosynthesis. The knock-out experiment in tobacco showed that *ycf2* gene was indispensable for plant cell survival and probably related to ATPase metabolic process [35]. The nucleotide sequences of *ycf2* were rich in diversity [36] and repeats [37]. Here, we retrieved two repeat copies in the *ycf2* gene, which were also shown in *Nicotiana tabacum* and *Arabidopsis thaliana*, indicating the essential structure in gene function [35].

Post-transcriptional control is important for the regulation of gene expression. The gene structures of introns and operons remained unknown, although some RNA editing sites were detected by using high-throughput RNA-seq [21]. Given the power of obtaining full-length transcripts without assembly from PacBio isoform sequencing (Iso-Seq), it is advantageous for gene annotation, identification of introns, RNA editing and operons in chloroplasts. An accurate and intact genome, as well as the well-defined annotation, will be beneficial to phylogenetic classification and to subsequently molecular studies.

### Introns and molecular evolution

Although an intron is a piece of non-coding DNA, there are many important implications for plant physiological activities and modern botanical applications. Introns are a group of self-catalytic ribozymes that could splice their own excision from mRNA, tRNA and rRNA precursors [38]. Introns help to infer phylogenetic relationships, better than the conserved genes such as *rbcL* due to their rapidly evolving noncoding sequences. Duckweeds represent the early-diverging monocot of the phylogenetic tree with their small and simple plant bodies, which is challenging to identify species by merely counting on morphology for non-experts. The method of DNA barcode of chloroplast markers alleviates such a situation by using PCR amplification and sequence variation. The overall polymorphisms of intergenic regions and introns are higher than the most coding DNA, providing valuable information to distinguish plant lineages. The *atpF*-*atpH* noncoding spacer was proposed as the best DNA barcoding marker for species-level identification of duckweeds [26]. Still, five out of 19 species failed to be separated from other sister species. Searching for more loci with enough variability would help to increase the discriminable resolution when they are combined with known markers. It was found that chloroplast introns showed the power of species identification with the sequence variability and the presence of highly conserved sequences in the flanking regions, which were suitable to design universal primers for DNA barcoding. The *ndhA* intron, together with the marker of *psbE-psbL* could distinguish *Fagopyrum* between species and subspecies [39]. Here, the comparison of nucleotide divergence and genetic distance between duckweed chloroplast coding sequences, intergenic regions and intron sequences offer scientists more markers to understand species phylogenetic relationship and plant evolution. Still, it is necessary to verify the potential of the utilization of *ndhA* intron itself or with other markers to distinguish intra- and inter-species in duckweeds.

### RNA editing and its evolution

RNA editing is a post-transcriptional modification that broadly exists in land plants from hornworts and ferns to seed plants. We could not detect RNA editing sites in the *Spirodela* chloroplast genome all at once only using one technique. With deep sequencing and various sequencing platforms, we expect more and more editing sites would be uncovered, especially for GC biased or very lowly expressed transcripts. Short reads generated by second-generation sequencing were able to define 66 editing sites [21]. Here, long reads using PacBio isoform sequencing identified 37 RNA editing sites. Excluding overlapped sites from two platforms, there are 74 RNA editing in *Spirodela*, more than twice of those in rice (35 sites) and maize (26 sites) [40]. The early-branching flowering plant of *Amborella trichopoda* was found to have 138 sites of RNA editing. It was proposed that early-branching flowering plants carried more abundant chloroplast RNA editing, whereas there was a tremendous decrease in RNA editing frequencies during flowering plant evolution [41]. To re-establish evolutionarily conserved amino acids and to maintain protein functions, *Spirodela*, as an early-diverging monocot shared many conserved editing sites with other plants, such as the *ndh* gene family of *ndhA*, *ndhB*, *ndhD* and *ndhF*. *Spirodela* also presents some species-specific RNA editing compared to its relatives, as some sites in *ndhB* [21].

### Operon and chloroplast photosynthetic reactor

In nuclear transgenic plants, the expression of multiple genes is time-consuming and extremely laborious with the requirement of putting one gene at a time and with subsequent backcrosses to select complete pathways with multi-subunit proteins, which is also compounded by variable expression levels. However, most chloroplast genes of plants are co-regulated and co-transcribed [42]. Such knowledge about operon structures would enable engineering new pathways in a simulated operon via a single transformation event into the chloroplast genome. It was reported that an artificially foreign pathway including seven genes was engineered into the tobacco chloroplast genomes [35]. Large amounts of foreign protein accumulation were observed in these transgenic lines, showing that the chloroplast posttranscriptional machinery can efficiently detect and translate genes in operons [43]. The *Bacillus thuringiensis* (Bt) cry2Aa2 operon was introduced into chloroplasts, resulting in 45% of the total soluble proteins in mature leaves and 100% of the observation of insect mortality after consuming the transgenic plants [44]. Understanding the operon information in duckweed chloroplasts lays the foundation and makes expressing foreign multiple proteins possible in terms of its rapid growth and biomass accumulation, facilitating duckweeds into an efficient photosynthetic reactor to produce pharmaceutical proteins or other foreign pathways.

## Conclusions

Here, a single circular strand genome with a size of 168,956 bp is directly constructed by using a long-read assembly without any manual correction and sequence collapses, skipping further PCR amplification to fill unassembled gaps. With the evidence of full-length cDNA generated from PacBio isoform sequencing, we accurately detect nine introns, 37 RNA editing sites and nine operons. We propose that the *ndhA* intron could be a potential species-barcoding marker with the sufficient genetic distance to discriminate close species, given its sequence divergence higher than the known *atpF-atpH* marker. In addition, the identified operon classes that encode the same functional complexes would lay the foundation of genetically engineering high protein, starch or oil duckweeds.

## Methods

### Plant DNA preparation and genome sequencing

*Spirodela polyrhiza* 7498 was originally collected by Dr. Elias Landolt from North Carolina, USA and kept in Wenqin Wang’s lab and Rutgers Duckweed Stock Cooperative (http://www.ruduckweed.org), which are publicly available. No permissions were necessary to collect the samples. The DNA was prepared from whole plant tissue using CTAB method [23]. *Spirodela polyrhiza* 7498 was sterilely cultured and fifty micrograms of high-molecular-weight total DNA was extracted. A 20-kb insert SMRTbell library was constructed and sequenced using the PacBio Sequel platform (Pacific Biosciences, Frasergen, Wuhan, China).

### PacBio isoform sequencing

The total RNA was isolated from the samples treated by multiple conditions (37 °C, 0 °C, desiccation, pH value of 9, UV exposure, 20 mg/l CuCl_2_, 300 mg/1 KNO_3_, 250 nM ABA, 10 mM kinetin, 300 mM mannitol) using TRIzol reagent (Invitrogen) and the RNeasy Mini kit after DNase I digestion (Qiagen). The extracted RNAs were evenly pooled. The library was constructed using a Clontech SMARTer PCR cDNA Synthesis Kit (Clontech) and sequenced on PacBio isoform sequencing (Iso-Seq) platform.

### Genome assembly and annotation

The PacBio raw reads were corrected into preads by Falcon (version 0.3.0) which was used in the downstream steps. The complete chloroplast genome of *Arabidopsis thaliana* (NC_000932.1) was downloaded from NCBI as a reference genome. The program BLASR (version 5.3.1) [45] was used to fish out the relevant chloroplast reads. The chloroplast genome of *S.polyrhiza* was assembled using a Perl-based software named “Organelle_PBA” (https://github.com/aubombarely/Organelle_PBA) [24]. The genome assembly pipeline generated by us was submitted to Github (https://github.com/Yating-zhang/chloroplast_Pacbio). The short-read and long-read based chloroplast genomes were aligned by BLASTN and the coverage plot was shown by using Samtools (version 1.7) [46]. The chloroplast genome was annotated with the tool GeSeq (https://chlorobox.mpimp-golm.mpg.de/geseq.html) [47] using the default parameters. The genome features were further manually curated by using transcript sequences to determine the boundary of introns and exons, and start codons as well. A circular map of the annotated genome was illustrated by using Organellar Genome DRAW (OGDRAW) (https://chlorobox.mpimp-golm.mpg.de/OGDraw.html) [48].

### Comparative genome analysis

The whole genome comparison between SpV1 and SpV2 was conducted by LASTZ alignment (version 1.02.00, http://www.bx.psu.edu/~rsharris/lastz/) and visualized in Geneious Prime® 2019.1.1. The chloroplast genomes of *Amborella trichopoda* (NC_005086.1), *Nicotiana tabacum* (NC_001879.2), *Arabidopsis thaliana* (NC_000932.1), *Oryza sativa Japonica Group* (NC_001320.1), *Zea mays* (NC_001666.2), *Lemna minor* (DQ400350), *Wolffiella lingulata* 7289 (JN160604) and *Wolffia australiana* 7733 (JN160604) were downloaded from NCBI GenBank and were aligned with SpV2 by Mauve (version 2.4.0) multiple genome alignment program.

### Intron, RNA editing and operon analysis

The full-length transcript generated by PacBio isoform sequencing were mapped to the previous and new chloroplast genome of *S.polyrhiza* by Gmap (version 2017-11-15) [49]. The RNA editing sites, introns and operons were identified through the mapped data and visualized under IGV (Integrative Genomics Viewer) (version 2.5.0) [50]. The four duckweed chloroplast genomes (*S.polyrhiza* (MN419335), *L.minor* (DQ400350), *W.ligulata* (JN160604) and *W*.*australiana* (JN160604)) were aligned and the genetic distance were computed by MEGA (version 6.06) [51]. The pairwise distance was calculated by the sequence polymorphism normalized by the sequence length.

## Supplementary information

**Additional file 1: Table S1.** Annotated gene list in the chloroplast of SpV2. **Table S2.** A comparison of detected introns among model plants. **Table S3.** The list of RNA editing sites in SpV2. **Figure S1.** Bioinformatic pipeline of chloroplast genome assembly and annotation. Details are described under Methods. **Figure S2.** The distribution of RNA editing events in the chloroplast genes of *S.polyrhiza*. Graph shows the number of currently detected RNA editing sites in protein coding genes. **Figure S3.** Alignments of editing sites in *rpoC2* and *ndhA* genes. The sequences included RNA editing sites are shown before RNA editing. The amino acid is in orange and substitutions are marked with arrows. The start, RNA editing and end locations are listed above the alignment. All aligned sequences are antisense from the reference except *rpoC2* and *rpoC1* gene of rice.

## Data Availability

The chloroplast genome assembly and annotation (SpV2) were deposited in GenBank under the accession number of MN419335.

## Abbreviations

BLAST: Basic local alignment search tool
PacBio: Pacific Biosciences
RNA-seq: RNA sequencing; Iso-Seq: isoform sequencing
SOLiD: Sequencing by Oligonucleotide Ligation and Detection
