# Supplementary material for: The complete chloroplast genome of greater duckweed (Spirodela polyrhiza 7498) using PacBio long reads: insights into the chloroplast evolution and transcription regulation
Source: BMC Genomics. 2020 Jan 28;21:76. doi: 10.1186/s12864-020-6499-y (PMC6986005; doi:10.1186/s12864-020-6499-y)
Supplement: Supplementary file 1 — Additional file 1: Table S1. Annotated gene list in the chloroplast of SpV2. Table S2. A comparison of detected introns among model plants. Table S3. The list of RNA editing sites in SpV2. Figure S1. Bioinformatic pipeline of chloroplast genome assembly and annotation. Details are described under Methods. Figure S2. The distribution of RNA editing events in the chloroplast genes of S.polyrhiza. Graph shows the number of currently detected RNA editing sites in protein coding genes. Figure S3. Alignments of editing sites in rpoC2 and ndhA genes. The sequences included RNA editing sites are shown before RNA editing. The amino acid is in orange and substitutions are marked with arrows. The start, RNA editing and end locations are listed above the alignment. All aligned sequences are antisense from the reference except rpoC2 and rpoC1 gene of rice. [file 12864_2020_6499_MOESM1_ESM.docx]

Supplementary Information

# TABLE S1. Annotated gene list in the chloroplast of SpV2.

| **Category** | **Gene group** | **Gene name** | | | | |
| --- | --- | --- | --- | --- | --- | --- |
| Self-replication | Ribosomal RNA genes | *rrn16* ^a^ | *rrn23* ^a^ | *rrn4.5* ^a^ | *rrn5* ^a^ |  |
|  | Transfer RNA genes | *trnH-GUG* | *trnY-GCU* | *trnS-GGA* | *trnP-UGG* | *trnA-UGC* ^a b^ |
|  |  | *trnQ-UUG* | *trnE-UUC* | *trnT-UGU* | *trnM-CAU* ^a^ | *trnS-AGA* ^a b^ |
|  |  | *trnS-GCU* | *trnT-GGU* | *trnF-GAA* | *trnL-CAA* ^a^ | *trnR-ACG* ^a^ |
|  |  | *trnR-UCU* | *trnS-UGA* | *trnD-GCC* | *trnV-GAC*^a^ | *trnN-GUU* ^a^ |
|  |  | *trnC-GCA* | *trnG-GCC* | *trnW-CCA* | *trnI-GAU* ^a b^ | *trnL-UAG* |
|  | Small subunit of ribosome | *rps16* ^b^ | *rps2* | *rps14* | *rps4* | *rps18* |
|  |  | *rps12* ^b d^ | *rps11* | *rps8* | *rps3* | *rps19* |
|  |  | *rps7* ^a^ | *rps15* ^a^ |  |  |  |
|  | Large subunit of ribosome | *rpl33* | *rpl20* | *rpl36* | *rpl14* | *rpl16* |
|  |  | *rpl22* | *rpl2* ^a b^ | *rpl23* ^a^ | *rpl32* |  |
|  | DNA-dependent RNA polymerase | *rpoC2* | *rpoC1* ^b^ | *rpoB* | *rpoA* |  |
| Photosynthesis | Subunits of photosystem I | *psaB* | *psaA* | *psaI* | *psaJ* | *psaC* |
|  | Subunits of photosystem II | *psbA* | *psbK* | *psbI* | *psbM* | *psbD* |
|  |  | *psbC* | *psbZ* | *psbJ* | *psbL* | *psbF* |
|  |  | *psbE* | *psbB* | *psbT* | *psbN* | *psbH* |
|  | Subunits of cytochrome b/f complex | *petN* | *petA* | *petL* | *petG* | *petB* |
|  |  | *petD* |  |  |  |  |
|  | Subunits of ATP synthase | *atpA* | *atpF* ^b^ | *atpH* | *atpI* | *atpE* |
|  |  | *atpB* |  |  |  |  |
|  | Subunits of NADH dehydrogenase | *ndhJ* | *ndhK* | *ndhC* | *ndhB* ^a b^ | *ndhF* |
|  |  | *ndhD* | *ndhE* | *ndhG* | *ndhI* | *ndhA* ^b^ |
|  |  | *ndhH* |  |  |  |  |
|  | Large subunit of Rubisco | *rbcL* |  |  |  |  |
| others | Maturase | *matK* |  |  |  |  |
|  | Envelope membrane protein | *cemA* |  |  |  |  |
|  | Subunit of acetyl-CoA | *accD* |  |  |  |  |
|  | C-type cytochrome synthesis gene | *ccsA* |  |  |  |  |
|  | Protease | *clpP* ^c^ |  |  |  |  |
|  | Genes of unknown function | *ycf3* ^c^ | *ycf4* | *ycf2* ^a^ | *ycf1* ^a^ |  |

^a^ *Two gene copies in IRs;* ^b^ *gene containing a single intron;* ^c^ *gene containing two introns;* ^d^ *gene divided into two independent transcription units.*

**TABLE S2.** A comparison of detected introns among model plants.

| **Gene** | **Species** | **Gene*** | **Intron*** | **Exon*** | **Intron_1*** | **Intron_2*** |
| --- | --- | --- | --- | --- | --- | --- |
| *ycf3* | *A.trichopoda* | 2084 | 1577 | 507 | 729 | 848 |
|  | *S.polyrhiza* | 2020 | 1513 | 507 | 746 | 767 |
|  | *O.sativa* | 1987 | 1477 | 510 | 745 | 732 |
|  | *Z.mays* | 1980 | 1467 | 513 | 736 | 731 |
|  | *A.thaliana* | 2008 | 1501 | 507 | 714 | 787 |
|  | *N.tabacum* | 2028 | 1521 | 507 | 738 | 783 |
| *clpP* | *A.trichopoda* | 2197 | 1588 | 609 | 842 | 746 |
|  | *S.polyrhiza* | 2065 | 1459 | 606 | 802 | 654 |
|  | *O.sativa* | 651 | 0 | 651 | 0 | 0 |
|  | *Z.mays* | 651 | 0 | 651 | 0 | 0 |
|  | *A.thaliana* | 1973 | 1382 | 591 | 867 | 515 |
|  | *N.tabacum* | 2035 | 1444 | 591 | 807 | 637 |
| *atpF* | *A.trichopoda* | 1825 | 1270 | 555 |  |  |
|  | *S.polyrhiza* | 1489 | 943 | 546 |  |  |
|  | *O.sativa* | 1371 | 828 | 543 |  |  |
|  | *Z.mays* | 1383 | 831 | 552 |  |  |
|  | *A.thaliana* | 1270 | 715 | 555 |  |  |
|  | *N.tabacum* | 1250 | 695 | 555 |  |  |
| *rpoC1* | *A.trichopoda* | 2764 | 721 | 2043 |  |  |
|  | *S.polyrhiza* | 2789 | 707 | 2082 |  |  |
|  | *O.sativa* | 2049 | 0 | 2049 |  |  |
|  | *Z.mays* | 2052 | 0 | 2052 |  |  |
|  | *A.thaliana* | 2834 | 791 | 2043 |  |  |
|  | *N.tabacum* | 2804 | 737 | 2067 |  |  |
| *rpl2* | *A.trichopoda* | 1482 | 652 | 830 |  |  |
|  | *S.polyrhiza* | 1484 | 663 | 821 |  |  |
|  | *O.sativa* | 1485 | 663 | 822 |  |  |
|  | *Z.mays* | 1485 | 663 | 822 |  |  |
|  | *A.thaliana* | 1507 | 682 | 825 |  |  |
|  | *N.tabacum* | 1491 | 666 | 825 |  |  |
| *rps12* | *A.trichopoda* | 903 | 531 | 372 |  |  |
|  | *S.polyrhiza* | 912 | 540 | 372 |  |  |
|  | *O.sativa* | 915 | 540 | 375 |  |  |
|  | *Z.mays* | 915 | 540 | 375 |  |  |
|  | *A.thaliana* | 909 | 537 | 372 |  |  |
|  | *N.tabacum* | 908 | 536 | 372 |  |  |
| *ndhA* | *A.trichopoda* | 2279 | 1187 | 1092 |  |  |
|  | *S.polyrhiza* | 2135 | 1052 | 1083 |  |  |
|  | *O.sativa* | 2076 | 987 | 1089 |  |  |
|  | *Z.mays* | 2112 | 1023 | 1089 |  |  |
|  | *A.thaliana* | 2163 | 1080 | 1083 |  |  |
|  | *N.tabacum* | 2240 | 1092 | 1148 |  |  |

**the length of nucleotides (bp) is determined by annotation files except the detected introns in SpV2.*

**TABLE S3.** The list of RNA editing sites in SpV2.

| **Genome Position** | **Gene** | **Amino Acid** | **Editing*** | **Coverage*** | **Efficiency (%)** |
| --- | --- | --- | --- | --- | --- |
| 11952* | non-coding | - | 62 | 236 | 26 |
| 15203* | atpF | P>L | 255 | 261 | 98 |
| 18456 | rps2 | T>I | 1069 | 1092 | 98 |
| 20135* | rpoC2-2 | S>F | 949 | 989 | 96 |
| 20639 | rpoC2-1 | S>L | 914 | 966 | 95 |
| 25833 | rpoC1-1 | P>L | 59 | 60 | 98 |
| 26691 | rpoB-2 | S>L | 33 | 33 | 100 |
| 28650 | rpoB-1 | S>L | 13 | 15 | 87 |
| 40491 | psbZ | S>L | 7 | 8 | 88 |
| 47241 | ycf3-5 | S>F | 30 | 41 | 73 |
| 48224 | ycf3-4 | P>L | 26 | 43 | 60 |
| 48230 | ycf3-3 | T>M | 26 | 43 | 60 |
| 48447 | ycf3-2 intron | - | 36 | 40 | 90 |
| 49098 | ycf3-1 | I>I | 21 | 34 | 62 |
| 72275 | petL | S>L | 3 | 3 | 100 |
| 85276 | rpoA | S>F | 228 | 240 | 95 |
| 86875 | rps8 | S>L | 227 | 237 | 96 |
| 90143 | rps3 | I>I | 83 | 110 | 75 |
| 90376 | rpl22 | S>L | 11 | 11 | 100 |
| 91211* | non-coding | - | 24 | 25 | 96 |
| 91213* | non-coding | - | 25 | 25 | 100 |
| 91215* | non-coding | - | 24 | 25 | 96 |
| 92921 | rpl23-2 | S>L | 8 | 10 | 80 |
| 92939 | rpl23-1 | S>F | 10 | 10 | 100 |
| 104732 | rps7-2 | A>A | 3 | 6 | 50 |
| 105777 | rps12 | S>L | 6 | 6 | 100 |
| 105884* | non-coding | - | 6 | 6 | 100 |
| 125342 | ndhF | S>L | 39 | 78 | 50 |
| 125447* | non-coding | - | 16 | 75 | 21 |
| 129488 | ndhD-2 | S>L | 4 | 4 | 100 |
| 130160 | ndhD-1 | T>M | 3 | 4 | 75 |
| 133695 | ndhA-5 | S>F | 351 | 407 | 86 |
| 133807 | ndhA-4 | P>S | 351 | 404 | 87 |
| 134193 | ndhA-3 | S>L | 228 | 391 | 58 |
| 135281 | ndhA-2 | P>L | 374 | 401 | 93 |
| 135335 | ndhA-1 | S>L | 340 | 341 | 100 |
| 135761 | ndhA | S>L | 151 | 393 | 38 |

**The number of reads. “Genome Position” shows the location of RNA editing in the chloroplast genome. Efficiency (%) is calculated the percentage of RNA editing by the edited reads divided by total mapped reads. “Amino Acid” is represented in abbreviation. “>” means the change of the amino acid. The eight newly defined editing sites are in green background.*


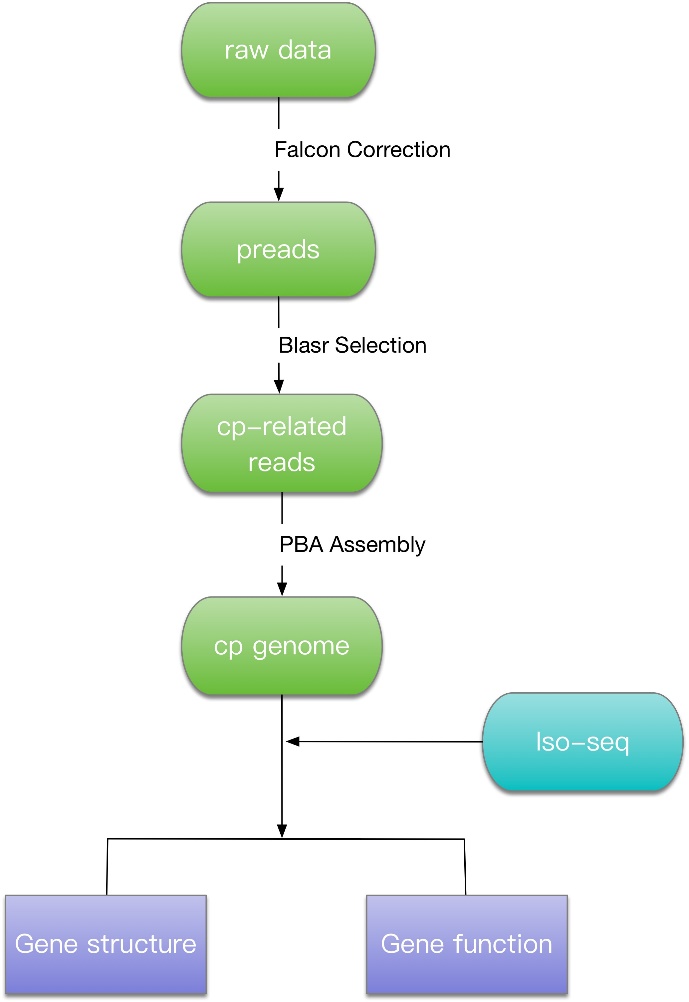


**Supplementary Figure 1.** Bioinformatic pipeline of chloroplast genome assembly and annotation. Details are described under Methods.


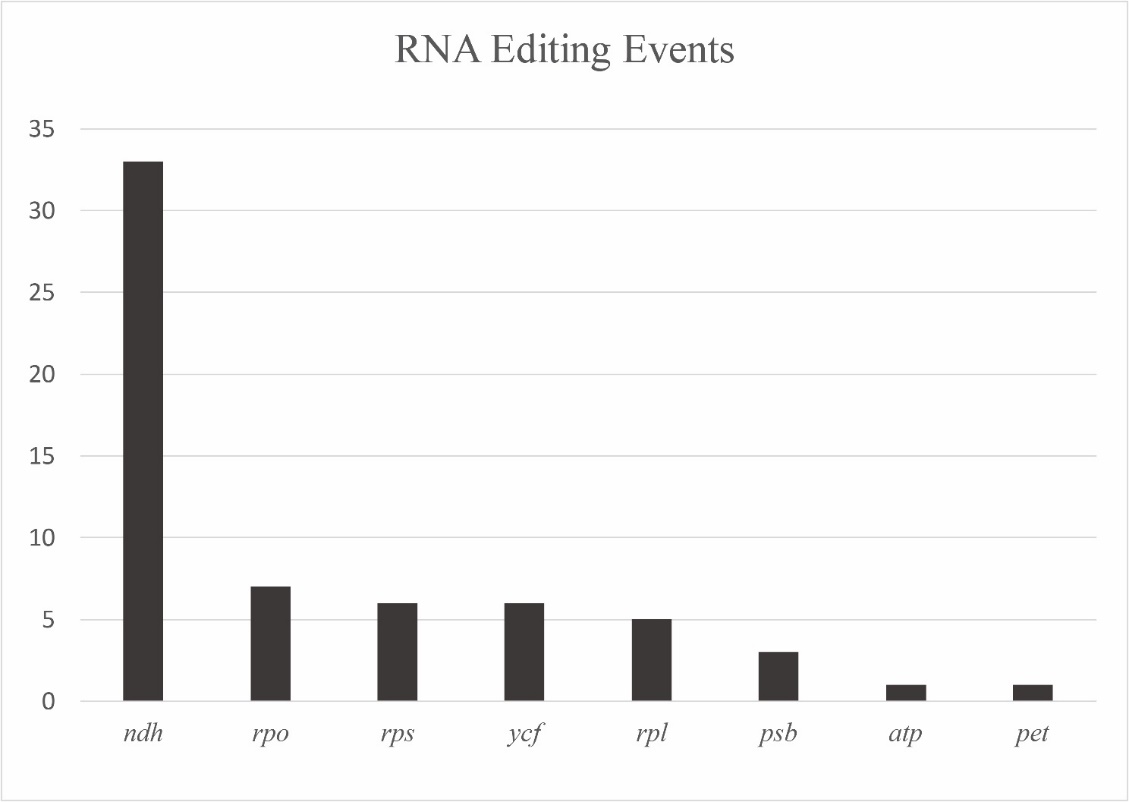


**Supplementary Figure 2.** The distribution of RNA editing events in the chloroplast genes of *S.polyrhiza*. Graph shows the number of currently detected RNA editing sites in protein coding genes.


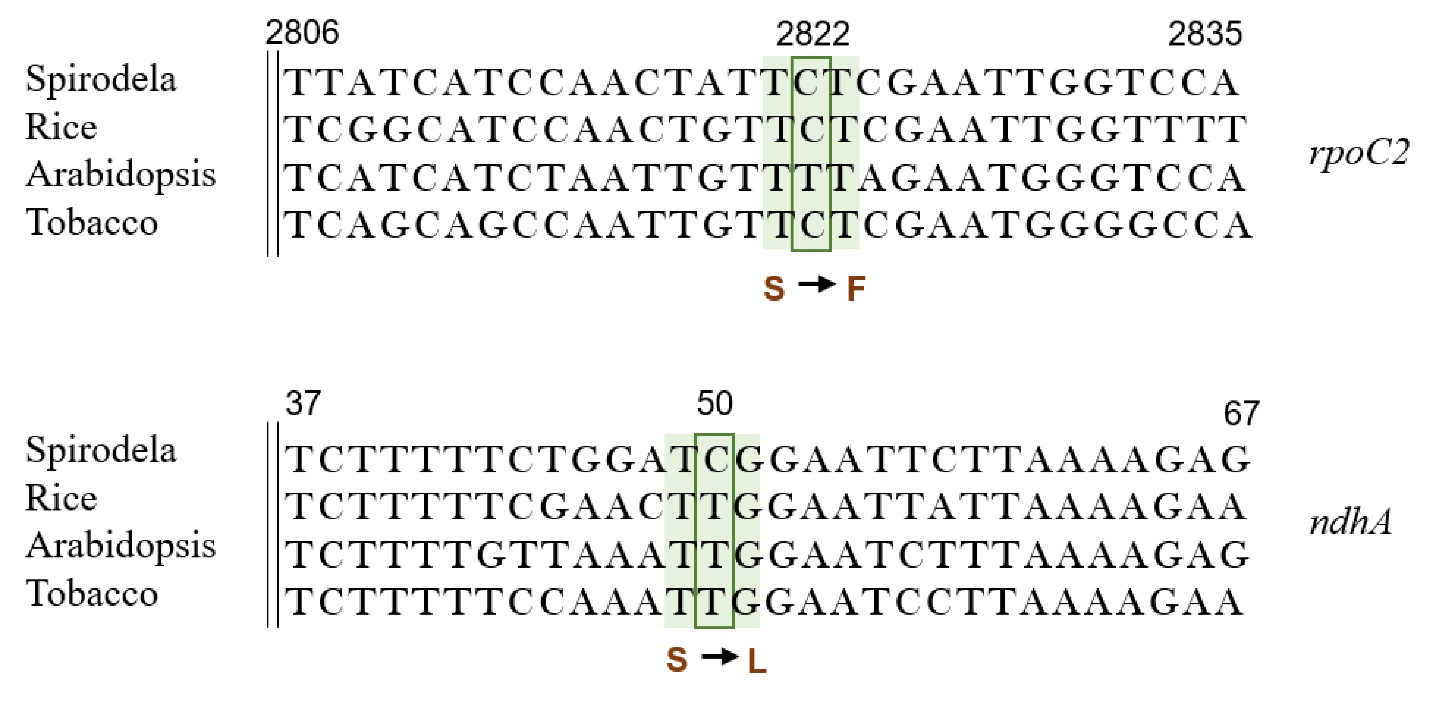


**Supplementary Figure 3.** Alignments of editing sites in *rpoC2* and *ndhA* genes. The sequences included RNA editing sites are shown before RNA editing. The amino acid is in orange and substitutions are marked with arrows. The start, RNA editing and end locations are listed above the alignment. All aligned sequences are antisense from the reference except *rpoC2* and *rpoC1* gene of rice.
